# Comparing plantar shear strain in patients with a previous diabetes‐related foot ulcer and those at low risk for ulceration using the STrain Analysis and Mapping of the Plantar Surface (STAMPS) system

**DOI:** 10.1111/dme.70164

**Published:** 2025-11-09

**Authors:** Alexander D. Jones, Sarah Crossland, Jane E. Nixon, Heidi J. Siddle, Peter R. Culmer, David A. Russell

**Affiliations:** ^1^ Leeds Vascular Institute Leeds Teaching Hospitals NHS Trust Leeds UK; ^2^ Leeds Institute of Clinical Trials Research University of Leeds Leeds UK; ^3^ Leeds School of Mechanical Engineering University of Leeds Leeds UK; ^4^ Leeds Institute of Rheumatic and Musculoskeletal Medicine University of Leeds Leeds UK

**Keywords:** diabetic foot, foot pressure

## Abstract

**Background:**

STrain Analysis and Mapping of the Plantar Surface (STAMPS) is an innovative system using a plastically deformable insole with a stochastic speckle pattern, developed to measure peak plantar shear strain (S_MAG_) in people with diabetes. The aim was to determine whether patients with a prior DFU exhibit higher S_MAG_ than low‐risk patients.

**Methods:**

Participants walked 20 steps with the STAMPS insole within a standardised shoe and 10 m with the Pedar‐X™ (Novel, Inc.) measurement insole. S_MAG_ was compared in participants with either a recently healed diabetic foot ulcer (Prior DFU group) or diabetes and low risk for ulceration (NICE NG‐19). Measurements were repeated three times. Images were analysed using the DIC software ‘GOM correlate’ (Zeiss, Inc.) and post‐processed using MATLAB. Outcomes were overall and regional peak S_MAG_ and peak plantar pressure (PPP). Consenting prior DFU participants subsequently repeated the walking assessments wearing a diabetic below‐knee walker‐boot. Overall and regional peak S_MAG_ and PPP were compared between the standard shoe and walker‐boot.

**Results:**

Twenty participants with prior DFU and 14 at low risk were recruited. Overall peak S_MAG_ within the prior DFU and low‐risk groups was 27.9% (IQR – 17.3–37.5%) and 11.5% (IQR 9.6–20.3%) respectively, *p* = 0.003. Within the prior DFU group, S_MAG_ was elevated at DFU sites compared with non‐DFU sites; peak S_MAG_ was 11.7% (IQR 7.6–25.6%) and 7.70% (4.4–13.1%). Sixteen participants completed the offloading assessments. Peak S_MAG_ within the standard shoe and walker‐boot was 27.4% (IQR 17.2–32.7) and 8.03% (IQR 6.3–12.2).

**Conclusion:**

Participants with a recently healed DFU exhibited elevated strain characteristics compared with the low‐risk group. Furthermore, prospective work will explore the relationship between S_MAG_ and DFU formation.

## 1 INTRODUCTION

An estimated 19%–34% of patients with diabetes will develop a diabetic foot ulcer (DFU). Within the first year of DFU development, 17% of patients will undergo a minor amputation and 5%–8% will undergo a major amputation.^1^, ^2^ Development of DFUs is driven by the interplay between peripheral neuropathy, foot deformity and peripheral arterial disease. Peripheral neuropathy and foot deformity result in pathological foot biomechanics and elevated plantar load. Plantar load comprises plantar pressure and plantar shear stress. A recent systematic review, conducted by the authors, highlighted the technical difficulty of shear assessment, demonstrated by the numerous methodologies implemented in its measurement.^3^ One group used a commercially available device,^4^ all other centres use custom‐made devices with significant variation in the technology used, including strain‐gauge systems,^5^, ^6^, ^7^, ^8^ piezoelectric transducers^9^ and magnetic resistive transducers.^10^ Furthermore, no devices have been developed that can measure both plantar pressure and plantar shear stress in‐shoe, across the plantar surface.^3^

**FIGURE 1:**
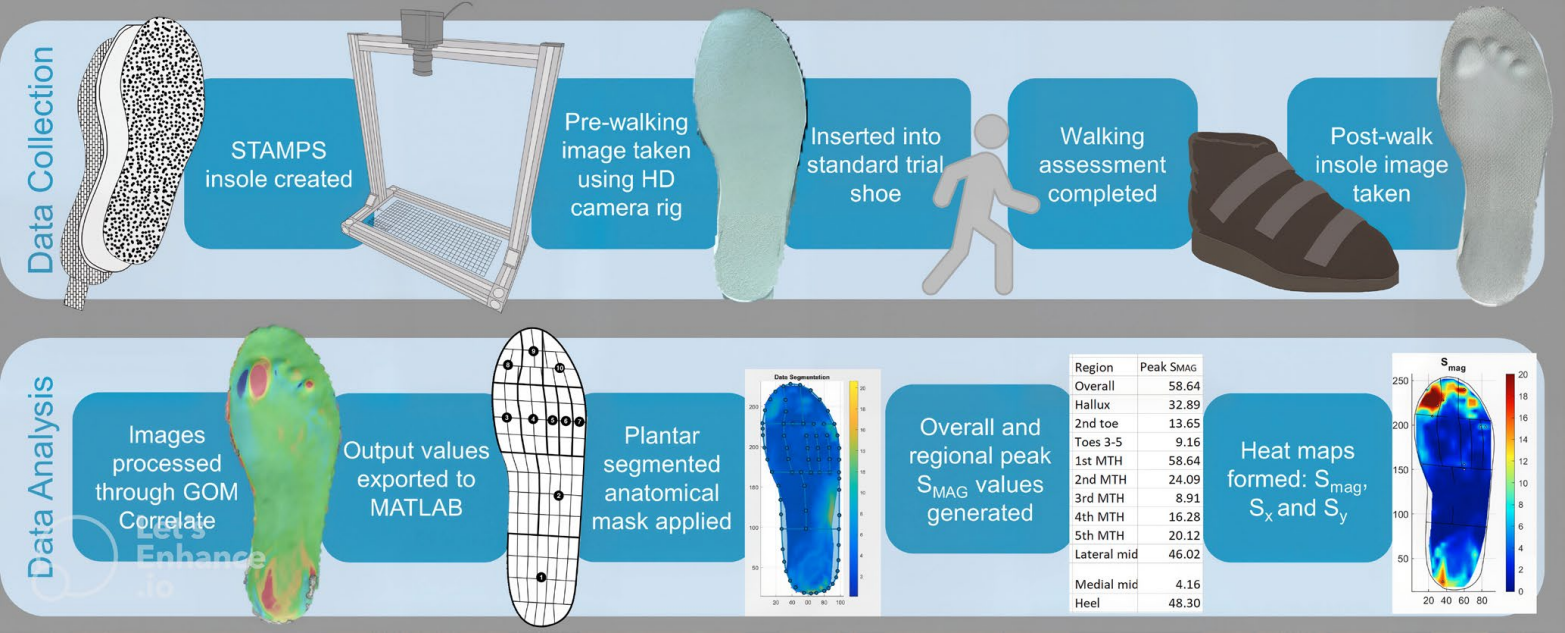
Overview of the STAMPS data collection and analysis process.

A greater understanding of plantar load and its role in DFU formation is required to improve DFU risk stratification and intervention strategies in patients with diabetes. To meet this requirement, the STrain Analysis and Mapping of the Plantar Surface (STAMPS) system was developed.^11^ STAMPS comprises a multilaminar, plastically deformable insole combined with digital imaging correlation (DIC) techniques to analyse plantar strain following a period of gait (Figure 1). A post‐walking image of the insole is compared with a pre‐walking image, and the pattern and magnitude of deformation are calculated using DIC software. The deformation is measured in the X and Y axes, with the X axis referring to medio‐lateral strain, and the Y axis antero‐posterior strain. The outcome of most interest is the resultant XY strain (S_MAG_).^11^ Strain is the change in size, or length of an object, relative to its original size following the application of force. In this context, strain is measured by comparing small areas (subsets) of the stochastic speckle pattern on the surface of the insole. Therefore, the strain metrics described are the percentage change, within the subsets, between the pre‐ and post‐ walking images, in the specified axes. These represent a measure of the cumulative effect of plantar pressure and shear stress at the foot–surface interface following a period of gait.

The technique has been described previously, and experimental testing validated the method to measure the effects of both vertical pressure and tangential shear stress at the foot–surface interface.^11^ It has subsequently been used to investigate the magnitude and patterns of strain in a healthy cohort.^12^ The primary aim of this study was to investigate whether people with a recently healed DFU exhibit elevated strain at the foot–surface interface compared with people at low risk for DFU formation. People at low risk for DFU formation were selected as the control group as it was expected that they would provide the most divergent comparator with regard to strain characteristics. In those with a previous DFU, it also aimed to investigate whether sites of previous DFU exhibit elevated strain and pressure compared with non‐DFU sites, to determine the prognostic precision of these measures for DFU location and to assess the efficacy of STAMPS to measure the effectiveness of offloading devices by comparing strain characteristics between the standard trial shoe and a diabetic below‐knee walker boot. Plantar pressure, measured by the Pedar‐X™ (Novel, Inc.) system, was used as a comparator.

## 2 METHODS

### 2.1 Study design

Ethical approval was obtained from the Health Research Authority; Research and Ethics Committee reference 22/YH/0007. This was a single centre case–control study. Cases consisted of people with a recently healed DFU (prior DFU group). The control (low‐risk) group consisted of people with diabetes at low risk of ulceration (NICE NG19 definition).^13^ The primary risk factor of interest was peak resultant strain (S_MAG_), measured using the STAMPS system. Plantar pressure analysis was performed using Pedar‐X™ to compare peak plantar pressure (PPP) between groups. To investigate the efficacy of the STAMPS system to measure the effectiveness of offloading devices, consenting participants also underwent STAMPS and Pedar‐X™ assessment within a diabetic below‐knee walker‐boot.

### 2.2 Participant recruitment

Based upon preliminary work, a mean difference in S_MAG_ of 4.5% was expected, with a standard deviation of 6%. Therefore 28 participants per group were required for 95% significance at a power of 80%. Due to a lower than anticipated recruitment rate, there was a failure to achieve the targeted 28 participants per group within the recruitment window. The reasons for this and its impact upon the results of the study are further addressed in the discussion. The 20 participants within the prior DFU group were recruited from the tertiary multidisciplinary diabetes limb salvage service (DLSS) at Leeds Teaching Hospitals NHS Trust. All patients with diabetes and a recently healed plantar DFU seen within the DLSS clinic were considered potentially eligible for this study. Patients included were ≥18 years of age with a diagnosis of diabetes mellitus; diabetic peripheral neuropathy (DPN); and a recently healed plantar DFU (healed within the last 3 months). Patients were excluded if they met any of the following exclusion criteria: those who are unable to mobilise independently for 150 m, without the use of walking aids and thus unable to complete the walking assessment; previous ipsilateral minor amputation; previous ipsilateral surgical off‐loading procedure; previous contralateral major amputation; ipsilateral toe pressure <40 mmHg; previous open or endovascular ipsilateral revascularisation. Sixteen participants consented to take part in the additional assessments using the diabetic below knee walker‐boot.

The 14 participants within the low‐risk group were recruited from the Leeds Community Healthcare NHS Trust Podiatry service. The service identified potentially eligible people who were sent a letter and the patient information leaflet. Potential participants were invited to contact the research team who then performed an eligibility assessment via telephone. People at low risk for DFU formation according to the NICE NG‐19 guidelines were included: people with diabetes whose only risk factor for DFU formation was callus alone. Patients were excluded if they met any of the following exclusion criteria: those who are unable to mobilise independently for 150 m, without the use of walking aids and thus unable to complete the walking assessments; toe pressure < 40 mmHg; presence of a foot deformity; previous contralateral major amputation.

### 2.3 Data collection

Demographics and clinical characteristics for each patient were documented. These characteristics are based upon reporting standards guidelines.^14^ History, examination and review of the medical records were performed to collect information on age, sex, weight, height, duration of diabetes, smoking status, duration of previous ulceration, time since previous ulceration, toe pressure, HbA1c (within the last 6 months), pre‐ulcerative lesion at entry, presence and type of foot deformity. The presence of co‐morbidities included chronic kidney disease, defined as an eGFR <60 for 3 months, and heart failure, based upon clinic records. For participants with multiple previous DFUs, only the most recently healed DFU was included. Diagnosis of DPN was made using the 10 g monofilament test.^15^ Foot deformity is defined as per the IWGDF: alterations or deviations from the normal shape or size of the foot, such as hammer toes, mallet toes, claw toes, hallux valgus, prominent metatarsal heads, pes cavus, pes planus, pes equinus, or results of Charcot neuro‐osteoarthropathy, trauma, amputations, other foot surgery or other causes.^16^

For the prior DFU group, the foot with the recently healed DFU was analysed. For the low‐risk group the right foot was selected. Evidence suggests that there is no difference in load characteristics between the dominant or non‐dominant limb; therefore, no bias should be introduced.^17^ To allow adjustment to an unfamiliar shoe, strain assessment was performed following a period of acclimatisation within the trial shoe and after pressure assessment.

The development of the STAMPS system has been described previously. However, the technique will be reviewed here in brief.^11^ Figure 1 demonstrates an overview of the STAMPS manufacture, data collection and analysis process. The STAMPS system comprises a multi‐layered, plastically deformable insole, the surface of which is covered with a stochastic speckle pattern. Pre‐ and post‐walking images are taken with a custom‐built digital camera platform (Ultra HD IMX317 USB camera, ELP Ltd) to obtain 4 K (3840 × 2160) images. Images are analysed using the DIC software ‘GOM correlate’ (Zeiss Inc.) before post‐processing occurs (MATLAB, Mathworks) using custom‐built analysis scripts.^18^

Participants' shoe size was measured and the appropriately sized supportive neoprene boot (Ninewells Boot, Chaneco) was used for both STAMPS and Pedar‐X™. This shoe was selected as the standard trial shoe as its volume allows the introduction of additional insoles. It also fully opens to the toes, facilitating insertion and removal of the STAMPS insole. A ‘pre’ image was taken of the insole's stochastic speckle pattern, which was inserted into the foot with prior DFU in the prior DFU group or the right shoe within the low‐risk group. A similarly sized insole was inserted into the contralateral shoe to prevent a discrepancy in insole depth. Participants were asked to walk 20 steps, along a flat surface, ensuring 10 steps were taken with the side of interest at a self‐selected normal walking speed. The insole was removed and a ‘post’ walking image of the insole's stochastic speckle pattern was taken. The time to traverse 5 m was recorded to assess gait speed. This process was repeated three times.

Participants within the prior DFU group opting in for the additional assessments completed the same walking assessments using the STAMPs insole whilst wearing a below‐knee walker‐boot (Rebound Airwalker, Steeper) on the side of prior DFU. A ‘pre’ image of the insole's stochastic speckle pattern was taken before insertion into the diabetic below‐knee walker‐boot. Participants were asked to walk 20 steps, along a flat surface at a self‐selected normal walking speed, ensuring 10 steps on the side of prior DFU. The insole was removed and a ‘post’ walking image of the insole's stochastic speckle pattern was taken. The time to traverse 5 m was recorded to assess gait speed. This process was repeated three times.

Pressure assessment was performed with the Pedar‐X™ in‐shoe plantar pressure measurement system. Participants were required to walk along the same flat surface, a distance of 10 m at their self‐selected normal walking speed.^19^ This process was repeated three times. Pressure assessment was performed on the side of the previous DFU within the prior DFU group and on the right foot within the low‐risk group. Participants within the prior DFU group opting in for the additional assessments completed walking assessments with the appropriately sized Pedar‐X™ insole within the below‐knee walker‐boot. These were completed using the same methodology as the standard trial shoe assessments.

### 2.4 Outcomes

- Primary
  ⚬ The difference in overall peak S_MAG_ between the prior DFU and low‐risk groups as measured by the STAMPS system
- Secondary
  ⚬ Peak S_MAG_ at sites of prior DFU compared with non‐DFU sites within the prior DFU group
  ⚬ Regional peak S_MAG_ compared between the prior DFU and low‐risk groups. Measured at the regions of the hallux, second toe, toes 3–5, 1st MTH, 2nd MTH, 3rd MTH, 4th MTH, 5th MTH, heel and medial and lateral midfoot
  ⚬ Overall and regional PPP compared between the prior DFU and low‐risk groups
  ⚬ Overall peak S_MAG_ compared between the standard trial shoe and below knee‐walker boot within the prior DFU group
  ⚬ Regional peak S_MAG_ compared between the standard trial shoe and below knee‐walker boot within the prior DFU group
  ⚬ Overall and regional PPP compared between the standard trial shoe and diabetic below knee walker‐boot
  ⚬ Inter‐observer reliability between a primary and secondary assessor for a subset of results
  ⚬ The relationship between peak S_MAG_ and PPP

### 2.5 Outcome definitions

Overall peak S_MAG_: The highest value of S_MAG_ across the whole plantar surface averaged across the three walking assessments of each participant.

Regional peak S_MAG_: The highest value of S_MAG_ within a region of the plantar surface averaged across each participant's three walking assessments.

Overall PPP: The highest value of plantar pressure across the whole plantar surface averaged across each participant's three walking assessments.

Regional PPP: The highest value of plantar pressure within a region of the plantar surface averaged across each participant's three walking assessments.

### 2.6 Statistical analysis

The Shapiro–Wilk test found the distribution of S_MAG_ deviated significantly from normal. Therefore, the median was used as a measure of central tendency. The Mann–Whitney U test was used to compare S_MAG_ between groups. Bivariable analysis using Spearman's correlation co‐efficient was used to investigate the relationship between potential co‐variates and S_MAG_. This was performed for the whole cohort of participants, as the relationships are expected to be constant irrespective of group. To adjust for covariates, ANCOVA was used to compare S_MAG_ between the groups. Peak S_MAG_ at the 20 prior DFU sites was compared with peak S_MAG_ at non‐DFU sites (i.e., all other regions) within the prior DFU group and all sites within the low‐risk group. Due to the significant positive skew of S_MAG_, one‐way non‐parametric ANOVA with Turkey's post hoc test was used to test statistical significance, results adjusted by Bonferroni correction for multiple comparisons. PPP at DFU sites were compared with non‐DFU sites within the prior DFU group and sites within the low‐risk group. One‐way ANOVA with Turkey's post hoc test was used to test statistical significance, results adjusted by Bonferroni correction for multiple comparisons. ROC curve analysis was used to assess the diagnostic precision of S_MAG_ and PPP to determine the site of previous DFU. The relationship between peak S_MAG_ and PPP was assessed with Spearman's correlation co‐efficient.

The difference in overall and regional S_MAG_ compared between the standard trial shoe and the diabetic below knee walker‐boot in the prior DFU group was calculated using the Hodges–Lehman test. The Wilcoxon signed rank test was used to test for statistical significance. The paired sample t‐test was used to compare overall and regional PPP between the standard trial shoe and the diabetic below knee walker‐boot. A sample of 15 participants' data sets was used to assess inter‐observer reliability of overall and regional peak S_MAG_, determined using intra‐class correlation co‐efficient (ICC).

## 3 RESULTS

Twenty participants were recruited within the prior DFU group, 14 participants within the low‐risk group.

**FIGURE 2:**
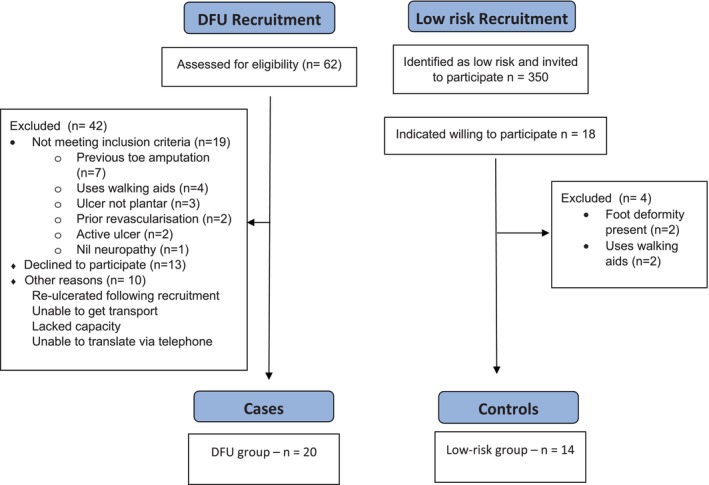
CONSORT flow diagram.

The CONSORT flow diagram is shown in Figure 2. Baseline characteristics are demonstrated in Table 1.

**TABLE 1:** Baseline characteristics. Means described with standard deviation in brackets.^16^

	Prior DFU (*n* = 20)	Low‐risk (*n* = 14)
Age	57.1 (10.1)	72.2 (12.9)
% Male	80.0	78.6
Weight (kg)	107.5 (20.9)	90.3 (15.8)
HbA1c (mmol/mol)	72.7 (19.7)	66.3 (11.4)
HbA1c (%)	8.8	8.2
Mean 5 m walk time standard trial shoe(s)	5.37 (1.7)	5.07 (0.83)
Mean 5 m walk time diabetic below knee walker‐boot (s)	5.69	
Current/Ex smoker (%)	45.0	64.2
Duration of diabetes (Months)	119.2 (114)	144.9 (123.5)
Toe pressure (mmHg)	127.6 (39.0)	119.2 (27.6)
Foot deformity^*^	7	
Previous ulcer location		
Hallux	7	
2nd toe	1	
Toes 3–5	2	
1st MTH	1	
2nd MTH	2	
3rd MTH	1	
4th MTH	1	
5th MTH	2	
Midfoot	0	
Heel	3	

^*^ Foot deformity defined as per the IWGDF ‐ Alterations or deviations from normal shape or size of the foot, such as hammer toes, mallet toes, claw toes, hallux valgus, prominent metatarsal heads, pes cavus, pes planus, pes equinus, or results of Charcot neuro‐osteoarthropathy, trauma, amputations, other foot surgery or other causes

### 3.1 Comparison of peak S_MAG_ and PPP between prior DFU and low‐risk groups

Peak S_MAG_ was significantly elevated within the prior DFU group compared with the low‐risk group; 27.9% (IQR 17.3%–37.5%) compared with 11.5% (IQR 9.6%–20.3%), respectively, *p* = 0.003. PPP was also significantly higher in the prior DFU group compared with the low‐risk group, 395.9 kPa (95% CI 357.4–434.6) versus 321.8 kPa (95% CI – 274.2–369.5 kPa), respectively, *p* = 0.015. Spearman's correlation co‐efficient for the comparison of age, walking speed and weight with S_MAG_ was −0.362, −0.226, and 0.475, respectively. Increased weight has been shown to be associated with increased PPP. Therefore, adjustment for weight was not performed, due to its known effect on increasing plantar load.^20^ To investigate whether the association between age and S_MAG_ was due to the age discrepancy between groups (i.e., negatively correlated with age due to the lower age within the low‐risk group) bivariable analysis was performed for each group. Spearman's correlation co‐efficient was 0.008 for the low‐risk group and − 0.252 for the prior DFU group, suggesting no relationship between S_MAG_ and age.

Regional comparison of peak S_MAG_ is shown in Table 2. A trend towards elevated peak S_MAG_ within the prior DFU group was found in each region, reaching significance in the regions of the 1st MTH and medial midfoot.

**TABLE 2:** Regional comparison of peak S_MAG_ between the prior DFU and low‐risk groups.

	Prior DFU		Low‐risk		*p*
	Peak S_MAG_ (%)	IQR (%)	Peak S_MAG_ (%)	IQR (%)	
Hallux	14.3	6.1–23.8	6.04	4.8–10.6	NS
2nd Toe	7.8	5.7–12.8	4.74	3.6–7.0	NS
Toes 3–5	8.1	5.6–15.0	5.2	3.2–10.4	NS
1st MTH	15.2	8.1–23.2	5.5	3.1–14.6	0.039
2nd MTH	11.0	6.0–13.0	5.2	3.9–12.4	NS
3rd MTH	6.2	3.6–8.1	3.3	2.4–6.4	NS
4th MTH	5.4	3.6–10.5	5.60	2.7–6.9	NS
5th MTH	6.4	3.6–12.2	7.0	4.0–8.3	NS
Midfoot Lateral	10.0	4.5–16.0	5.8	2.6–8.2	NS
Midfoot Medial	4.1	3.3–5.9	2.3	1.8–5.1	0.043
Heel	10.6	8.5–18.1	9.11	7.5–10.3	NS

### 3.2 Relationship between DFU location, S_MAG_ and PPP

Within the prior DFU group, peak S_MAG_ was located in the same region as the previous DFU in 30% of participants, PPP was located in the same region as the previous DFU in 35% of participants. Table 3 demonstrates the comparison of peak S_MAG_ and PPP between DFU and non‐DFU sites within the prior DFU group and the low‐risk group. A moderate correlation was found between S_MAG_ and PPP, *r* = 0.526.

**TABLE 3:** Peak S_MAG_ and PPP at DFU sites and non‐DFU sites within the prior DFU group and the low‐risk group.

		Median (%)	IQR (%)	*p*
All Sites	Low‐risk	5.7	3.1–8.9	*p* < 0.001 vs. Non‐DFU/prior DFU
	Non‐DFU	7.7	4.4–13.1	*p* = 0.037 versus prior DFU
	Prior DFU	11.6	7.6–25.6	
Hallux and lesser toes	Low‐risk	5.7	3.9–10.5	*p* = 0.004 vs. prior DFU
	Non‐DFU	8.4	5.4–15.5	*p* = 0.165 vs. prior DFU
	Prior DFU	16.5	7.7–29.5	
MTHs	Low‐risk	5.2	3.1–8.8	NS
	Non‐DFU	7.3	4.1–13.0	NS
	Prior DFU	11.3	4.5–18.0	
Heel	Low‐risk	9.1	7.4–11.0	NS
	Non‐DFU	10.7	8.9–18.1	NS
	Prior DFU	7.7	NA	

		Mean (kPa)	95% CI (kPa)	*p*
All Sites	Low‐risk	171.9	158.8–185.0	*p* = 0.04 vs. Non‐DFU, *p* < 0.001 vs. prior DFU
	Non‐DFU	196.6	182.5–210.8	*p* = 0.01 vs. prior DFU
	Prior DFU	262.0	207.6–316.3	
Hallux and lesser toes	Low‐risk	158.1	134.4–181.8	*p* = 0.01 vs. prior DFU
	Non‐DFU	162.2	135.1–189.3	*p* = 0.02 vs. prior DFU
	Prior DFU	249.4	165.4–333.4	
MTHs	Low‐risk	201.4	184.1–218.7	*p* = 0.05 vs. prior DFU
	Non‐DFU	226.1	207.8–244.5	*p* = 0.23 vs. prior DFU
	Prior DFU	280.4	179.1–381.7	
Heel	Low‐risk	233.0	198.6–267.3	NS
	Non‐DFU	289.7	242.3–337.2	NS
	Prior DFU	260.8	155.93–677.6	

### 3.3 Relationship between DFU location, S_MAG_ and PPP–ROC curve analysis

**FIGURE 3:**
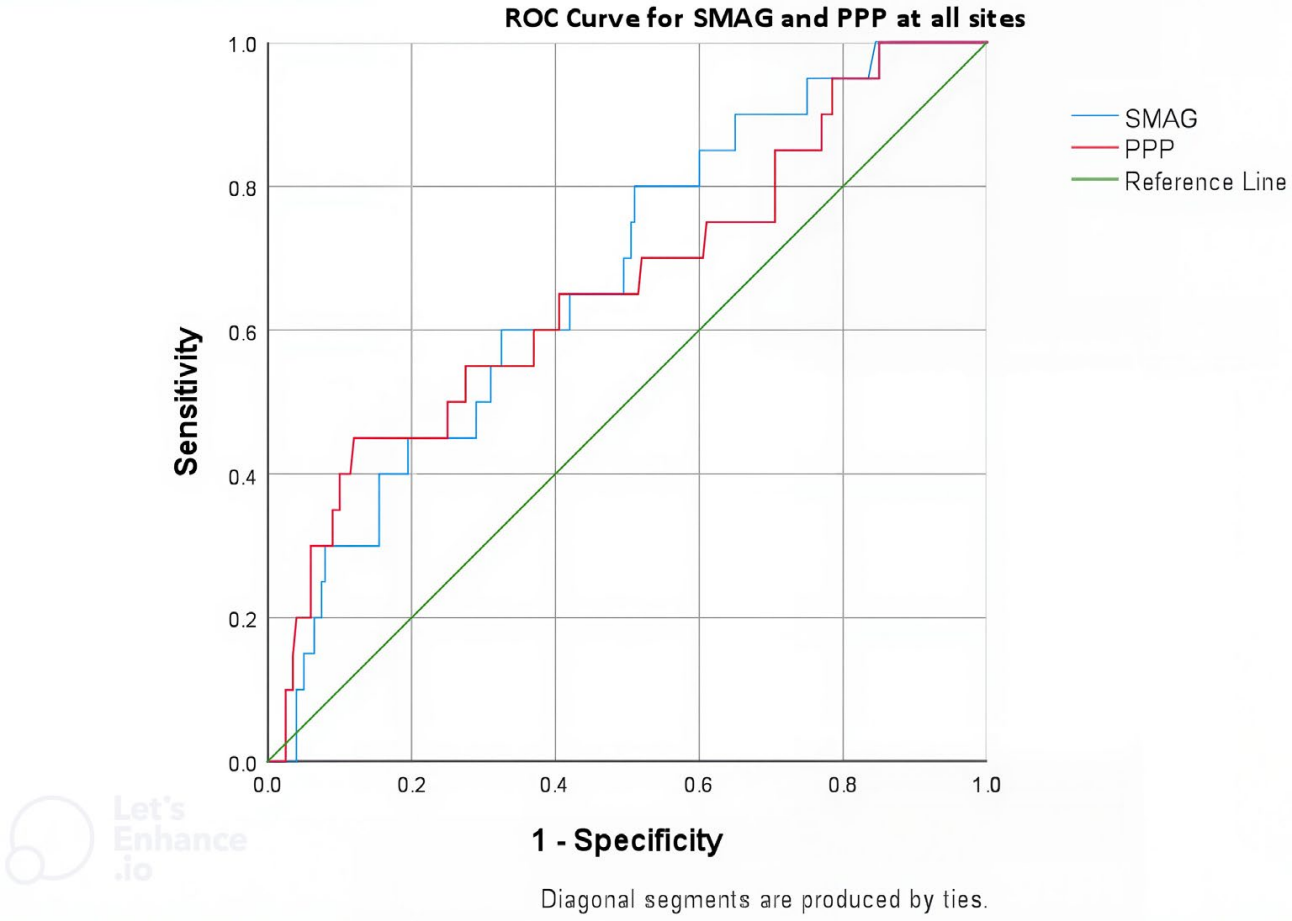
ROC curve analysis for diagnostic precision of site‐specific S_MAG_ and PPP for DFU location.

ROC curve analysis was used to investigate the predictive value of site‐specific S_MAG_, PPP and ulcer location (Figure 3), both ROC curves deviated significantly from 0.5. Utilising S_MAG_, the area under the ROC curve was 0.67 (95% CI: 0.56–0.79). The optimum S_MAG_ threshold of 11% had a sensitivity of 60% and a specificity of 67.5% for predicting prior DFU. Utilising PPP, the area under the ROC curve was 0.66 (95% CI: 0.53–0.79). Using a S_MAG_ threshold of 11% resulted in a positive likelihood ratio of 1.8 and a negative likelihood ratio of 0.59. Using the optimum PPP of 220 kPa resulted in a positive likelihood ratio of 1.6 and a negative likelihood ratio of 0.64.

### 3.4 Comparison of peak S_MAG_ between the standard trial shoe and diabetic below knee walker‐boot in the prior DFU group

**FIGURE 4:**
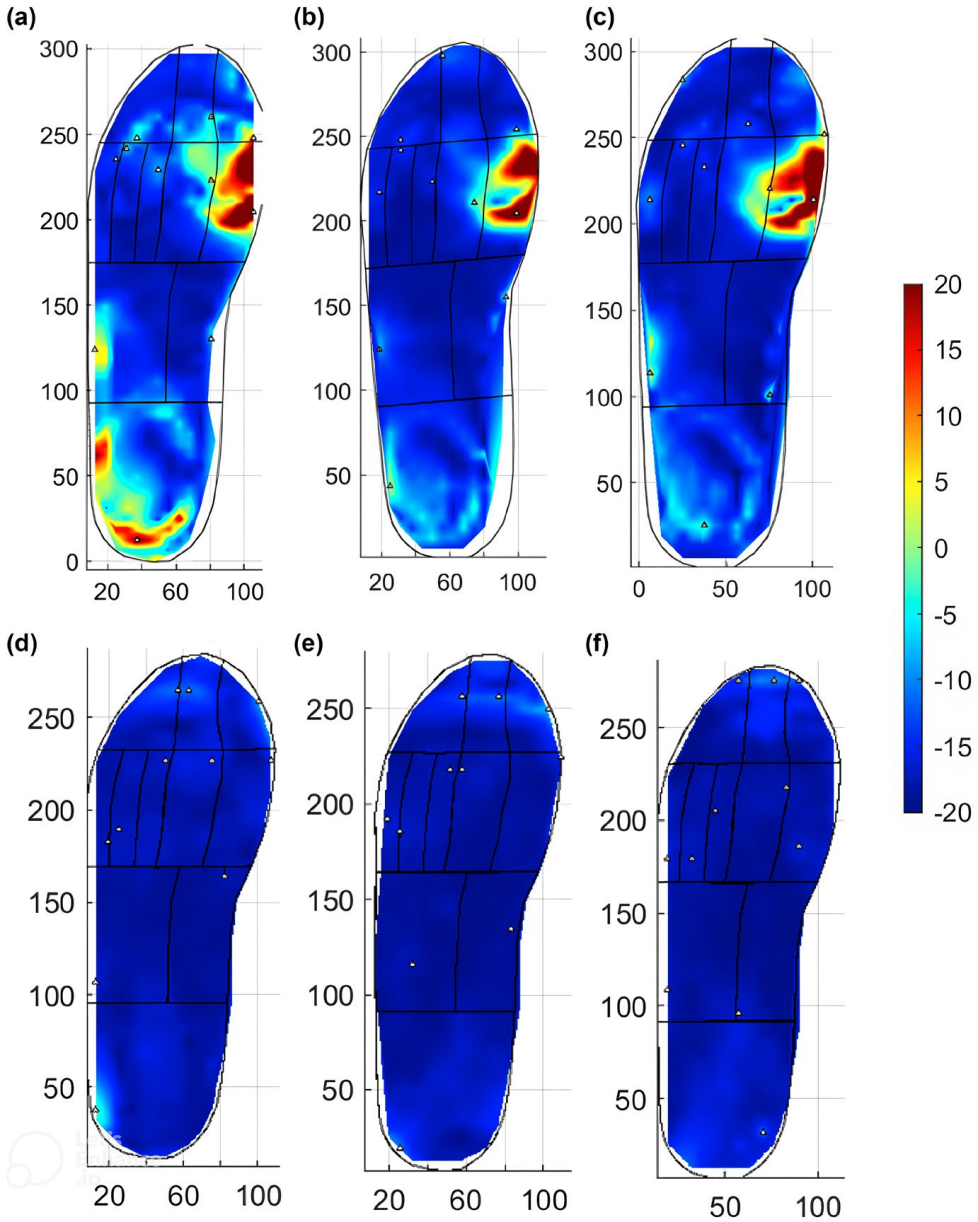
S_MAG_ heat plots of participant 15's standard shoe walking assessments (a–c) and diabetic below knee walker‐boot assessments (d–f).

Sixteen participants completed the walking assessments with a diabetic below‐knee walker‐boot. Overall peak S_MAG_ was significantly lower whilst wearing a diabetic below‐knee walker‐boot compared with the standard trial shoe; 8.0% (IQR 6.4–12.2) and 27.4% (IQR 17.1–32.7), median difference 14.1%, 95% CI 8.8–20.5%, *p* < 0.001. Overall PPP was significantly lower whilst wearing a diabetic below‐knee walker‐boot compared with the standard trial shoe, 184.7 kPa (95% CI 163.0–206.5) and 393.1 kPa (95% CI 349.4–436.8) respectively, *p* < 0.001. Mean difference 208.3 kPa (95% CI 168.7–247.9), *p* < 0.001. Figure 4 shows Participant 15's S_MAG_ patterns compared between the standard trial shoe and the below‐knee walker‐boot.

### 3.5 Inter‐observer reliability

A single measure, absolute‐agreement two‐way random effects model was used. The ICC was 0.988, 95% CI: 0.979–0.993. This suggests excellent inter‐observer reliability.^21^

## 4 DISCUSSION

The primary aim of this was to investigate whether people with a recently healed DFU exhibit elevated plantar strain at the foot–surface interface compared with those at low risk of DFU. The results demonstrated significantly elevated overall peak S_MAG_ between the prior DFU and low‐risk groups. A significant association was identified between regional S_MAG_ and DFU location and regional PPP and DFU location. Sites of previous DFU exhibited significantly elevated S_MAG_ compared with non‐DFU sites, within the prior DFU group. PPP was significantly elevated at DFU sites, compared with non‐DFU sites. Though both demonstrated only modest discrimination (area under the curve 0.67 and 0.66 respectively), S_MAG_ was equivalent to PPP in determining the location of prior DFU. Prior studies have found the location of PPP to be a poor predictor of DFU location.^22^, ^23^ Ledoux et al. found elevated site‐specific plantar pressure to be associated with DFU formation only at the MTHs.^22^ Abbott et al. used a novel approach to barefoot plantar pressure assessment and found it to have modest diagnostic precision for site‐specific DFU; area under the curve of 0.7.^24^ More recently the concept of cumulative plantar tissue stress and its association with DFU formation has been explored. Cumulative plantar tissue stress describes the accumulation of all stresses on the foot: pressure, shear stress, weight‐bearing activity, and use of offloading footwear.^25^ Hulshof et al., found no association between elevated cumulative plantar tissue stress and DFU formation, though identified slower walking speed and pre‐ulcerative lesions as predictive factors.^26^ The evidence supports the biomechanical model for DFU formation, though no single measure of plantar load is strongly predictive of DFU formation.^27^ Within the present study, the prior DFU group comprised patients with a recently healed DFU; therefore, the associations identified are not indicative of DFU development but rather related to prior ulceration. Prospective studies of people with diabetes at risk of first DFU are required to further explore the relationship between elevated plantar strain and DFU development.

Peak S_MAG_ was significantly reduced when participants completed the walking assessments in the diabetic below‐knee walker‐boot compared with the standard trial shoe. A reduction in peak S_MAG_ and PPP was found in almost all anatomical regions in the diabetic below‐knee walker‐boot compared with the standard shoe. These results suggest the STAMPS system has the potential to be utilised to assess the effectiveness of offloading devices. Previous studies have demonstrated diabetic below‐knee walker‐boots can achieve a PPP reduction of 50–85%, in line with the results of the present study.^27^, ^28^ The change in PPP was higher in most regions compared with S_MAG_, most notably in the toes and lateral MTHs. This may indicate that despite pressure offloading, a degree of shear stress remains causing S_MAG_ to be elevated compared with PPP.

Limitations are present within this study. This was a case–control study, designed as a proof of concept to investigate whether people with a prior DFU exhibit elevated strain compared with people at low‐risk for DFU formation. Selection bias was present; cases comprised high‐risk patients with a prior DFU, controls were low‐risk, thus the groups differed in factors other than DFU history. Further work will be undertaken in patients with DPN, with and without ulceration to further investigate the relationship between strain and DFU. To reduce selection bias, matching for weight was considered though not performed. Previous work has demonstrated that increased weight is positively correlated with increased PPP.^29^ Matching for weight would potentially match a factor associated with elevated plantar load, therefore it was not performed. It was hypothesised that both age and walking speed may be associated with S_MAG_ and PPP. However, no such associations were identified. To reduce selection bias, cases were recruited consecutively from a multidisciplinary diabetes limb salvage clinic. Inclusion and exclusion criteria were also considered to reduce selection bias; patients with prior amputation were excluded to reduce heterogeneity between groups patients with significant peripheral arterial disease were also excluded. Comparing S_MAG_ at DFU and non‐DFU locations inherently introduces bias and likely overestimates the true effect, however this analysis is consistent with other studies and allows better comparison amongst the literature.^22^, ^24^

This study has a small sample size and failed to meet the recruitment target for both groups; under recruiting by eight participants within the prior DFU group and 14 within the low‐risk group. This resulted in a smaller than expected sample size, and impacts the conclusions that can be drawn, however, it should be noted, the original power calculation was based upon earlier iterations of the STAMPS design, with different analysis methods. Despite under recruitment, the effect size was greater than anticipated, resulting in a significant primary end‐point difference. Several factors drove under recruitment; many patients with healed ulcers were excluded due to previous amputation, previous revascularisation and the use of walking aids. Furthermore, it was a challenge for many patients to attend the study assessment, either due to lack of time or transport. Future studies will undertake strain assessment within the clinic appointment, obviating the need to return, which would better reflect potential clinical practice.

Despite national screening strategies, 60,000 people in the United Kingdom present with a new DFU each year.^30^ Between five and 8% of these will go on to have a major amputation within one year, 10% will die, and even after healing, 40% will reoccur within 1 year.^2^ The population‐level evidence demonstrates how current prevention and treatment strategies are failing to tackle the problem. Management strategies for both primary prevention, secondary prevention and ulcer healing revolve around plantar pressure redistribution. However, plantar pressure comprises only part of the load sustained by the plantar surface of the foot. Using only pressure as a marker for risk, or as a target for intervention may result in the failure to identify and mitigate against a key factor in DFU formation. Using STAMPS to assess and address plantar load in its entirety may form part of the solution; however, prospective work is required to investigate the relationship between S_MAG_ and DFU formation. The second aspect warranting investigation is whether offloading guided by the STAMPS assessment reduces the risk of DFU formation. Further work is also undertaken to ameliorate the technique of plantar strain assessment. The current system performs two‐dimensional DIC; a three‐dimensional system to measure deformation in the z‐axis is under development. In addition, analytical methods to derive the applied stresses from the measured strain are being developed.

## 5 CONCLUSION

This study demonstrates that patients with a recently healed DFU exhibit elevated S_MAG_ compared with people at low risk for ulceration. Furthermore, prospective work is required to investigate the possible association between elevated S_MAG_ and DFU formation.

## AUTHOR CONTRIBUTIONS

**Peter Culmer:** Writing – review & editing, Supervision, Software, Resources, Methodology, Investigation, Funding acquisition, Formal analysis, Conceptualisation. **Heidi Siddle:** Writing – review & editing, Supervision, Methodology. **David A. Russell:** Writing – review & editing, Supervision, Resources, Methodology, Investigation, Funding acquisition, Conceptualisation. **Sarah Crossland:** Writing – review & editing, Software, Project administration, Methodology, Formal analysis, Data curation. **Alexander D. Jones:** Writing – original draft, Validation, Project administration, Methodology, Investigation, Formal analysis, Data curation, Conceptualisation. **Jane Nixon:** Writing – re view & editing.

## FUNDING INFORMATION

The research is supported by the National Institute for Health Research (NIHR) infrastructure at Leeds. The views expressed are those of the author(s) and not necessarily those of the NHS, the NIHR or the Department of Health and Social Care. The authors would like to thank the NIHR MedTech and In Vitro diagnostics Co‐operatives (MICs) and NIHR BRC at Leeds. Mr. David Russell is supported in part by the National Institute for Health and Care Research (NIHR) Leeds Biomedical Research Centre (BRC) (NIHR203331) and an NIHR Advanced Fellowship (NIHR300633). Dr. Heidi Siddle, Senior Clinical Lecturer, ICA‐SCL‐2018‐04‐ST2‐004, is funded by Health Education England (HEE)/National Institute for Health Research (NIHR) for this research project. The views expressed in this publication are those of the author(s) and not necessarily those of the NIHR, University of Leeds, NHS or the UK Department of Health and Social Care. Dr. Sarah Crossland: Part of this research was supported by an EPSRC funded CDT Centre Grant EP/L01629X/1.

## CONFLICT OF INTEREST STATEMENT

The authors declare no conflict of interest.
